# Hypokalemia in patients with anorexia nervosa during refeeding is associated with binge–purge behavior, lower body mass index, and hypoalbuminemia

**DOI:** 10.1186/s40337-021-00452-2

**Published:** 2021-08-06

**Authors:** Michitaka Funayama, Yu Mimura, Taketo Takata, Akihiro Koreki, Satoyuki Ogino, Shin Kurose, Yusuke Shimizu

**Affiliations:** 1 Department of Neuropsychiatry, Ashikaga Red Cross Hospital, 284-1 Yobe, Ashikaga-city, Tochigi 326-0843 Japan; 2 Department of Neuropsychiatry, Keio University School of Medicine, Shinjuku, Tokyo Japan; 3 Department of Neuropsychiatry, National Hospital Organization Shimofusa Psychiatric Medical Center, Chiba, Japan; 4 Department of Trauma and Critical Care Medicine, Kyorin University School of Medicine, Mitaka, Tokyo Japan

**Keywords:** Hypokalemia, Body mass index, Albumin, Binge–purge behavior, Refeeding, Anorexia nervosa

## Abstract

**Background:**

Hypokalemia is frequently found in patients with anorexia nervosa and sometimes leads to life-threatening conditions. Although their serum potassium levels are considered to further decrease during refeeding, no previous studies have addressed actual changes in the serum potassium levels and potential mechanisms underlying hypokalemia during the refeeding period of patients with anorexia nervosa. In this study, we investigated factors associated with hypokalemia during refeeding of patients with anorexia nervosa.

**Methods:**

We recruited 52 independent patients from 89 admissions with anorexia nervosa (body mass index, 13.0 ± 3.3) from the psychiatry unit in Ashikaga Red Cross Hospital during the period from April 2003 to March 2018 and analyzed serum potassium levels at admission. Of the 89 admissions, 66 admissions with > 1-week hospitalization were recruited to determine the lowest potassium levels during the refeeding period. We analyzed these levels with multiple linear regression analysis with explanatory variables, including data upon admission and treatment-related indicators.

**Results:**

The initial serum potassium level of 3.6 ± 0.9 mg/dl decreased to 3.1 ± 0.7 mg/dl at nadir hypophosphatemia, which was observed an average of 2.5 days after admission. A lower serum potassium level at admission and a lower nadir potassium level during refeeding were associated with a lower body mass index, hypoalbuminemia, and binge–purge behavior. Similar results were obtained when the analysis included restrictive or binge–purge types as well as the independent patient group.

**Conclusions:**

Lower body mass index, hypoalbuminemia, and binge–purge behavior might be used as indicators to guide clinical approaches for controlling serum potassium levels in patients with anorexia nervosa during refeeding.

**Plain English summary:**

Hypokalemia, low levels of serum potassium, in patients with anorexia nervosa sometimes leads to life-threatening conditions. Thus, it is of great importance to predict the risk of hypokalemia in patients with anorexia nervosa during the refeeding period. Our study found that hypokalemia in patients with anorexia nervosa during refeeding is associated with a lower body mass index and hypoalbuminemia (low levels of serum albumin), in addition to binge–purge behavior.

## Introduction

Hypokalemia is frequently observed in patients with anorexia nervosa [1–3]. Some patients with anorexia nervosa may be able to adapt to severe chronic hypokalemia [4]. In other patients, however, hypokalemia can lead to life-threatening conditions, e.g., QTc-interval prolongation [5], ventricular fibrillation [6, 7], torsades de pointes [8], acute kidney injury [9], and interstitial nephritis [10, 11]. Regarding mechanisms responsible for hypokalemia, binge–purge behavior and laxative/diuretic abuse are believed to induce hypokalemia [3, 12] via gastrointestinal fluid loss and the associated renal loss of potassium [13]. Other potential mechanisms underlying hypokalemia in patients with anorexia nervosa include severe malnutrition and its associated low potassium intake [12, 14, 15], induction by hypomagnesemia [16, 17], and surges in insulin that occur during the refeeding process [1, 18–20].

Inpatients with anorexia nervosa presumably have a higher risk of developing hypokalemia because they suffer from severe malnutrition and are refed [21], in which the insulin surge resulting from glycemia during the refeeding process causes a substantial intracellular uptake of potassium and phosphorus [20]. Potassium is the major intracellular cation and is taken up into cells as the cells increase in volume and number with the change to anabolism upon refeeding and as a direct result of insulin secretion, even though potassium had been depleted as a result of malnutrition in these individuals. Thus, their serum potassium levels are considered to further decrease during refeeding. No previous studies, however, have addressed actual changes in the serum potassium levels and potential mechanisms underlying hypokalemia during the refeeding period of patients with anorexia nervosa.

It is crucial for clinicians to predict the severity of hypokalemia during the refeeding period because the potassium nadir is usually observed after patients are refed, suggesting that there might be life-threatening conditions not only at admission but also during refeeding [22]. Thus, in this study, we carried out a retrospective investigation of mechanisms behind hypokalemia in patients with anorexia nervosa by using laboratory data at admission as well as treatment-related indicators during the refeeding period.

## Materials and methods

### Participants

**Fig. 1:**
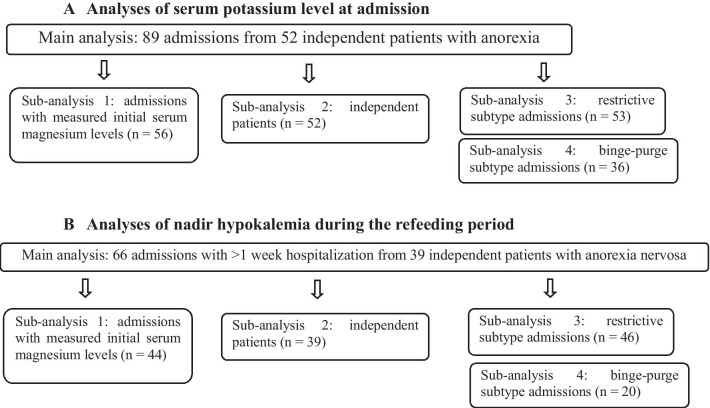
**A** Analyses of serum potassium level at admission. **B** Analyses of nadir hypokalemia during the refeeding period

Ethical aspects of this study were reviewed and approved by the Human Research Ethics Committee at the Ashikaga Red Cross Hospital. This study was performed after obtaining informed consent from all participants upon admission. For patients below the age of 18 years, informed parental consent was also obtained. Diagnosis was based on criteria in the ICD-10, and each patient was diagnosed by two of the three psychiatrists, each of whom is a board certified specialist for psychiatry and had > 10 years of experience in psychiatry at the time of the study. Participants were recruited from the neuropsychiatric unit in Ashikaga Red Cross Hospital during the period from April 2003 to March 2018, during which there were 90 admissions with anorexia nervosa (F50.0) that were managed in our unit. These were categorized into the restrictive type (F50.01, anorexia nervosa, restricting type) and the binge–purge type (F50.02, anorexia nervosa, binge eating/purging type), again by two of the three psychiatrists. One patient was excluded because her serum albumin levels at admission, an indicator of morbidity that leads to malnutrition [23], were not examined. Thus, 89 admissions met the above mentioned criteria and were included in this study for serum potassium levels at admission (Fig. 1A). Consecutive admissions with recurrences of anorexia nervosa were included as separate admissions [24–26] because weight and nutritional status change with each admission [24]. In this study, among a total of 52 patients, all of whom were Japanese, 16 had two or more consecutive admissions, which added up to a total of 89 admissions.

Of the 89 admissions, 66 admissions that involved hospitalization for > 1 week were used to investigate for nadir hypokalemia. This is because refeeding syndrome, in particular electrolyte imbalance, typically occurs within the first week of refeeding [25–27], and because the effect of refeeding on serum potassium levels is the main focus of this study. Among a total of 70 admissions with hospitalization for > 1 week, data from four admissions were excluded because second blood tests were not carried out within 6 days after admission for these individuals: three patients declined repeated blood tests, and one patient underwent the second blood test 8 days after admission. Thus, 66 admissions from 39 independent patients met the above mentioned criteria and were included in this study for the nadir hypokalemia level (Fig. 1B).

### Collection of patient information

Electronic medical records of eligible participants were retrospectively reviewed. As outcome indicators, the following two measures were used: serum potassium levels at admission and nadir potassium levels during the first 2 weeks after admission. Although electrolyte imbalance during the refeeding period typically occurs within the first week of refeeding [25–27], refeeding hypophosphatemia occasionally occurs more than one week after admission and recovers approximately two week after admission [25]. Indeed, in our present study, nadir hypokalemia was observed during the second week in 11 admissions (16.6%). Thus, in this study, interval of the first 2 weeks was applied for the lowest potassium levels. Explanatory variables included data at admission, i.e., age, sex, body mass index, anorexia nervosa subtype (restrictive or binge–purge), data obtained from laboratory tests (serum albumin level, blood urea nitrogen/creatinine ratio, and serum magnesium levels), and indicators involving treatment, i.e., the rate of weight gain during the first 7 days, caloric intake, and amount of potassium administered. Serum magnesium values were corrected for serum albumin using this formula: albumin-corrected magnesium = magnesium + 0.005 (4.0 – albumin g/dl) [28, 29]. Classification of anorexia nervosa subtype, restrictive or binge–purge (bulimic-type), was carried out because binge–purge behavior, as well as its accompanying laxative/diuretic abuse, often contributes to lower serum potassium levels through repeated vomiting and diarrhea and its associated renal potassium loss in the urine [3, 12, 13]. Body mass index, serum albumin level, and blood urea nitrogen/creatinine ratio were included as explanatory variables because those characteristics have been repeatedly identified as indicators for the severity of refeeding hypophosphatemia [24–26, 30, 31]. A lower body mass index reflects malnutrition itself [23] while a lower serum albumin level indicates morbidity that leads to malnutrition [23]. Causes for a higher blood urea nitrogen/creatinine ratio include mainly volume depletion, but also protein-energy malnutrition and the catabolic state, and elevated corticosteroid levels, all of which are closely related to the malnutrition found in patients with anorexia nervosa [26, 32, 33]. Indeed, all p-values of the correlations between each of the three variables and serum potassium level at admission were less than 0.1. In contrast, serum creatinine level, an indicator for muscle mass as well as renal function and its related hyperkalemia, was excluded from the explanatory variables because there was no relationship between serum creatinine levels and serum potassium levels at admission (p = 0.91). Body mass index was calculated as the weight of the individual (in kilograms) divided by the square of the height of the individual (in meters). The indicators involving treatment were used because those factors might affect refeeding syndrome [20, 26]. To calculate the rate of weight gain during the first 7 days, we divided the kilograms gained during the first 7 days by body weight at admission. Total caloric intake (kilocalories) refers to the average total caloric intake from day 1 through day 7 [25, 26], including both oral intake and intravenous infusion therapy. If the patient ate only half the provided 1200-kcal meal, the actual amount of total caloric intake was recorded as 600 kcal. To accurately investigate the effect of energy intake on an individual patient depending on his or her weight, an indicator of total caloric intake per body weight at admission was used for this analysis (total caloric intake divided by body weight at admission), which is widely used for diet therapy for diabetes mellitus [34]. Potassium administration was defined as the average daily total potassium administration (intravenously and orally combined) from day 1 through day 7 (milliequivalents, mEq). To better calculate the effect of potassium supplementation, the amount of potassium administered was divided by body weight at admission, and this value was used in the statistical analysis.

A laboratory panel, including serum potassium levels, was carried out on admission. Regarding the 66 admissions used for the measurement of nadir hypokalemia, each blood test from the second examination onwards was conducted at 7:30 in the morning before breakfast. To precisely identify nadir serum potassium levels, the patients frequently underwent serial laboratory tests: 48 admissions (72.7%) were tested on the second hospital day, 43 (65.1%) on the third hospital day, 43 (65.1%) on the fourth hospital day, 34 (51.5%) on the fifth hospital day, 34 (51.5%) on the sixth hospital day, 29 (43.9%) on the seventh hospital day, and 28 (42.4%) on the eighth hospital day. These patients continued to have blood tests until their serum potassium levels went up again. We note that 60 of 66 admissions (90.9%) had the second laboratory test within 72 h of the first.

### Protocol for refeeding and potassium administration

The initial caloric prescription for each patient was decided by individual physicians on admission, based on their assessment of the degree of malnutrition, caloric intake preceding admission, and the weight of each patient. Although caloric intake was administered mainly through oral food (meals and liquid formulas with nutrient compositions), intravenous infusion therapy was sometimes used and, less frequently, nasogastric feeding (liquid formulas with nutrient compositions) was also carried out. Normally, the total initial caloric prescription consisted of ~ 600–1400 kcal/day and was usually increased by ~ 200 kcal every day. Potassium supplementation more than 20 mEq per day was not routinely carried out. However, it was prescribed when hypokalemia (less than 3.5 mEq/L) was found in the laboratory test at admission or in subsequent tests. Intravenous potassium administration was carried out mainly with a normal sugar electrolyte maintenance transfusion solution (10 mEq in a 500-ml transfusion) or an intravenous potassium infusion of ~ 20–30 mEq in 500-mL transfusion solution bags. In the acute phase, potassium administration was prescribed intravenously, whereas oral potassium administration was also carried out, in particular, in the subacute phase. The amount of potassium administration was generally started with ~ 20–40 mEq per day, which was adjusted depending on the levels of serum potassium on serial laboratory tests. This procedure was similarly applied for hypophosphatemia and hypomagnesemia [20].

### Statistical analysis

Serum potassium level at admission was investigated from data of 89 admissions (52 independent patients) (Fig. 1A), whereas data from an additional 66 admissions (39 independent patients) with hospitalization for > 1 week were used to determine the nadir hypokalemia during refeeding (Fig. 1B). Multiple linear regression analysis was used with explanatory variables, including demographics (age, sex, body mass index, and anorexia nervosa subtype, i.e., restrictive or binge–purge), laboratory data obtained at admission (serum albumin level, blood urea nitrogen to creatinine ratio, and albumin-corrected magnesium level), and treatment-related indicators (caloric intake, amount of potassium administered, and rate of weight gain). The number of patients with laxative/diuretic abuse was 20 according to the electronic medical records, all of whom were included in the binge–purge group, which poses a risk for multicollinearity in the multiple linear regression analysis. The exact number of those patients might have been higher because patients with anorexia nervosa tend to hide such abuse, and a structured interview in a prospective study is needed to precisely investigate it. For these reasons, laxative/diuretic abuse was not included as an independent explanatory variable in this retrospective study, and it was included with the binge–purge behavior. No single variable had a correlation of > 0.52 with other variables, indicating that all variables were relatively independent, such that all variables were included in the following analyses.

For the analysis of serum potassium level at admission, six explanatory variables were used, including age, sex, anorexia nervosa subtype, body mass index, blood urea nitrogen to creatinine ratio, and serum albumin level, whereas the treatment-related variables (caloric intake, amount of potassium administered, and rate of weight gain) as well as the above mentioned six explanatory variables were also used for analysis of nadir hypokalemia during the refeeding period. Sub-analyses were also repeated using the four additional cohorts (Fig. 1A, B): patients with measured initial serum magnesium levels (sub-analysis 1), the group of independent patients (sub-analysis 2), the participants with restrictive type (sub-analysis 3), and those with binge–purge type (sub-analysis 4). Because serum magnesium levels were not measured during the night shift in this facility until December 2013, with the exception of certain special cases, we repeated the analyses using 56 admissions for serum potassium levels at admission (Fig. 1A, sub-analysis 1) and 44 admissions for nadir potassium levels (Fig. 1B, sub-analysis 1), all of whom had undergone complete blood tests including determination of initial serum magnesium levels and albumin-corrected magnesium level was include in this analysis. Analyses using only independent patients were conducted because of a potential bias related to consecutive admissions (Sub-analysis 2). The first admission for each individual patient was included in this cohort: 52 independent patients for initial serum potassium levels (Fig. 1A, sub-analysis 2) and 39 independent patients for nadir hypokalemia during refeeding (Fig. 1B, sub-analysis 2). To take into account the potential heterogeneity among participants with anorexia nervosa depending on the disorder subtype, we carried out separate comparisons among data for individuals with the restrictive subtype (sub-analysis 3 with 53 admissions) and for those with the binge–purge subtype (sub-analysis 4 with 36 admissions) for each explanatory variable using Student’s t test and Fisher’s exact test. The above mentioned multivariable analyses were also performed for each subtype. Regarding the group of patients with binge–purge subtype (sub-analysis 4), all of them were female and the explanatory variable of sex was excluded. Given that the hallmark of refeeding syndrome is refeeding hypophosphatemia [19, 20], the correlation between a decrease in serum phosphorus levels and in potassium levels during refeeding was also investigated.

Excel 2010 (Microsoft, Redmond, WA, USA) with add-on Statcel 3 (OMS Ltd., Tokyo, Japan) was used for all statistical analyses. Two-tailed p-values are reported, and p-values of < 0.05 were considered statistically significant.

## Results

### Demographic factors, laboratory data at admission, and treatment indicators

Table 1 shows the demographic factors, laboratory data at admission, and treatment indicators for the study group. The data represents mean ± standard deviation. The average age was 34.2 ± 11.2 years (range: 14 to 59 years). Of 89 admissions, only four (4.5%) were male participants. No patient had medical conditions that might involve hypokalemia or hyperkalemia, including primary hyperaldosteronism, Cushing syndrome, hyperthyroidism, renal tubular acidosis, Bartter syndrome, Gitelman’s syndrome, Addison’s disease, or moderate or severe chronic renal disease, all of which were excluded by the criteria for each medical condition that was established by experts in each field in Japan. Likewise, none received antihypertensive drugs, mineralocorticoids, Kanzou (a traditional Chinese medicine), or insulin therapy, all of which affect serum potassium levels. Body mass index was relatively low at 13.0 ± 3.3 (range: 7.9–21.2). The average total caloric intake during the first 7 days was 1237 ± 582 kcal/day. Although the average serum potassium level at admission was 3.6 ± 0.9 mmol/l (range: 1.7–5.8 mmol/l), there was a significant difference between subtypes (Student’s t-test, p < 0.01): the average serum potassium level at admission for individuals with the restrictive type was 4.0 ± 0.7 mmol/l, whereas that for individuals with the binge–purge type was 3.2 ± 0.9 mmol/l. The average nadir potassium level was 3.1 ± 0.7 mmol/l (range: 1.7–4.4 mmol/l) with 3.3 ± 0.6 mmol/l for individuals with the restrictive type and 2.9 ± 0.8 mmol/l for individuals with the binge–purge type, levels that were also significantly different (Student’s t-test, p < 0.01). The time interval between admission and nadir hypokalemia was longer for individuals with the restrictive type with 3.3 ± 3.4 days when compared with individuals with the binge–purge type with 1.3 ± 3.0 days (Student’s t-test, p < 0.01). In a total of 11 admissions (16.6%), 10 with the restrictive type and 1 with the binge–purge type, nadir hypokalemia was observed during the second week after admission. In sub-analysis 1, there was a mild negative correlation of albumin-corrected magnesium level with serum potassium level at admission (r = − 0.39, p < 0.01). In fact, albumin-corrected magnesium levels were influenced by prehospital prescription of magnesium oxide, a laxative. The albumin-correlated magnesium level of patients with prescription of magnesium oxide (n = 5) was 3.2 ± 1.1 mg/dl, which was higher than that of patients without prescription of magnesium oxide (n = 51, 2.0 ± 0.4 mg/dl) (Mann–Whitney U test, p < 0.01). Of five patients who had been prescribed with magnesium oxide before being hospitalized, three were categorized into the binge–purge type. When these five patients were excluded, there was no significant correlation of albumin-corrected magnesium level with serum potassium level at admission (n = 51, p = 0.07).

**Table 1:** Demographic factors, laboratory data at admission, and treatment indicators of admissions with anorexia nervosa (N = 89)

Characteristic	Total patients (N = 89)	Patients with restrictive type (n = 53)	Patients with binge–purge type (n = 36)	p value for comparisons between types (Student’s t test, except as noted)
**Demographics**				
Age (years)	34.2 ± 11.2	37.8 ± 11.0	28.9 ± 9.4	< 0.01
Sex (female participants)	95.5%	92.5%	100%	0.14 (Fisher’s exact test)
**Data at admission**				
Weight (kg)	33.3 ± 9.0	31.6 ± 6.4	35.7 ± 11.5	0.03
Body mass index	13.0 ± 3.3	12.3 ± 2.2	14.1 ± 4.3	0.01
Albumin (g/dl)	3.9 ± 0.7	3.8 ± 0.7	4.0 ± 0.6	0.18
Blood urea nitrogen/creatinine ratio	29.8 ± 18.5	35.3 ± 20.7	21.6 ± 10.5	< 0.01
Serum potassium level (mmol/l)	3.6 ± 0.9	4.0 ± 0.7	3.2 ± 0.9	< 0.01
Serum phosphorus level (mg/dl)	3.9 ± 1.3	3.7 ± 1.4	4.1 ± 1.0	0.18
Albumin-corrected magnesium (mg/dl) (56 cases)	2.1 ± 0.6	2.0 ± 0.4 (36 cases)	2.4 ± 0.8 (20 cases)	0.02
**Caloric intake and weight gain**				
Total caloric intake during the first 7 days (kcal/day)	1237 ± 582	1221 ± 646	1269 ± 428	0.75
Weight gain during hospitalization (kg)	2.1 ± 2.6	2.4 ± 2.6	1.7 ± 2.6	0.18
Data during refeeding period*				
Nadir serum potassium level (mmol/l)	3.1 ± 0.7	3.3 ± 0.6	2.9 ± 0.8	< 0.01
Time interval between admission and nadir hypokalemia (days)	2.5 ± 3.4	3.3 ± 3.4	1.3 ± 3.0	0.01
Potassium administration during the first 7 days (mEq/day)	24.1 ± 22.7	19.2 ± 13.3	33.9 ± 34.5	0.01
Weight gain during the first 7 days (kg)	1.1 ± 2.0	0.8 ± 1.8	1.9 ± 2.0	0.03
Nadir serum phosphorus level (mg/dl)	2.7 ± 0.8	2.5 ± 0.8	3.2 ± 0.7	< 0.01

Data represents mean ± standard deviation

*Data from 66 admissions with hospitalization of > 1 week, consisting of 46 admissions of patients with restrictive anorexia nervosa and 20 admissions of patients with anorexia nervosa, binge eating/purging type

### Serum potassium level at admission

The multiple linear regression model showed that the binge–purge type (p < 0.001), serum albumin level (p < 0.01), and body mass index (p = 0.01) predicted the serum potassium level at admission (Table 2). This model resulted in a p-value of < 0.001 and F-value of 7.8, which is statistically significant, and explained 36.2% of the observed variance. When only the 56 admissions for which serum magnesium levels at admission were included (Fig. 1A, sub-analysis 1), the results were similar with binge–purge type (p < 0.001) and body mass index (p = 0.01) influencing the serum potassium level at admission (p < 0.001, F = 9.0). When the analysis was repeated with only the 52 independent patients (Fig. 1A, sub-analysis 2), the binge–purge type (p = 0.01), serum albumin level (p = 0.02), and body mass index (p = 0.03) again predicted the serum potassium level at admission (p < 0.001, F = 4.6). When data from only patients with the binge–purge type were considered (Fig. 1A, sub-analysis 4), the multiple linear regression model again showed that body mass index (p = 0.04) predicted the serum potassium level at admission (p < 0.01, F = 4.6). When this analysis was repeated only with data from patients with the restrictive type (Fig. 1A, sub-analysis 3), however, the model did not reach statistical significance (p = 0.16). In sum, the three variables, i.e., binge–purge behavior, a lower body mass index, and a lower serum albumin level, were associated with a lower serum potassium level at admission in patients with anorexia nervosa.

**Table 2:** Multiple linear regression analysis for serum potassium level at admission among patients with anorexia nervosa

Characteristic	p value	Standard beta	Regression coefficient	Standard error	Lower 95% confidence limit	Upper 95% confidence limit
**Binge–purge subtype**	**7.450 × 10**^**−06**^	**− 0.493**	**− 0.908**	**0.190**	**− 1.286**	**− 0.531**
**Albumin**	**0.008**	**0.263**	**0.353**	**0.129**	**0.097**	**0.610**
**Body mass index**	**0.011**	**0.267**	**0.074**	**0.029**	**0.017**	**0.131**
Blood urea nitrogen/creatinine ratio	0.100	0.167	0.008	0.005	− 0.002	0.018
Age	0.637	− 0.050	− 0.004	0.009	− 0.021	0.013
Sex	0.931	0.008	0.035	0.404	− 0.768	0.839

Items with the bold formatting are statistically significant factors for serum potassium level at admission.

### Nadir hypokalemia during refeeding

The multiple linear regression model showed that the binge–purge type (p = 0.02), lower caloric intake (p = 0.03), and a lower serum albumin level (p = 0.04) predicted a lower nadir hypokalemia during refeeding (Table 3). Although the relationship between body mass index and nadir hypokalemia was not statistically significant, body mass index has a tendency to affect the nadir potassium level (p = 0.07). This model resulted in a p-value of < 0.001 and F-value of 4.7, which is statistically significant, and explained 42.5% of the observed variance. When only the 44 admissions for which serum magnesium levels at admission were included (Fig. 1B, sub-analysis 1), the results were similar with binge–purge type (p < 0.001), lower body mass index (p = 0.02), and lower caloric intake (p = 0.04) predicting a lower nadir hypokalemia level (p < 0.001, F = 6.0). When the analysis was repeated with only the 39 independent patients (Fig. 1B, sub-analysis 2), the binge–purge type (p = 0.03) was associated with the nadir hypokalemia (p < 0.01, F = 3.0). When data from only patients with the restrictive type were considered (Fig. 1B, sub-analysis 3), the model (p = 0.02, F = 2.7) showed that lower caloric intake (p = 0.04) predicted a lower nadir potassium level, with albumin having a tendency to affect nadir hypokalemia (p = 0.08). When the analysis was repeated using data from only participants with the binge–purge type (Fig. 1B, sub-analysis 4), the model did not reach statistical significance (p = 0.38). In sum, a lower nadir potassium level during refeeding was associated mainly with the four variables: binge–purge type, a lower body mass index, a lower serum albumin level, and lower caloric intake.

**Table 3:** Multiple linear regression analysis for nadir potassium level during refeeding

Characteristics	p value	Standard beta	Regression coefficient	Standard error	Lower 95% confidence limit	Upper 95% confidence level
**Binge–purge subtype**	**0.021**	**− 0.291**	**− 0.449**	**0.189**	**− 0.828**	**− 0.070**
**Caloric intake**	**0.033**	**0.250**	**0.008**	**0.004**	**0.001**	**0.015**
**Albumin**	**0.040**	**0.233**	**0.253**	**0.120**	**0.012**	**0.493**
Body mass index	0.070	0.231	0.073	0.039	**− **0.006	0.151
Potassium administration	0.124	**− **0.187	**− **0.136	0.087	**− **0.311	0.039
Weight gain	0.130	**− **0.174	**− **0.019	0.012	**− **0.043	0.006
Age	0.635	**− **0.058	**− **0.004	0.008	**− **0.021	0.013
Blood urea nitrogen/creatinine ratio	0.673	0.053	0.002	0.005	**− **0.007	0.011
Sex	0.683	0.048	0.198	0.483	**− **0.768	1.165

Items with the bold formatting are statistically significant factors for a nadir potassium level

### Relationship between hypophosphatemia and hypokalemia during refeeding

Among 66 admissions with hospitalization of > 1 week, the extent of the decrease in the serum phosphorus level from admission to nadir hypophosphatemia was 1.1 ± 1.4 mg/dl, whereas that of the serum potassium level was 0.4 ± 0.5 mmol/l. A moderate positive correlation was found between the two electrolytes (r = 0.35, p < 0.01). Among participants with the restrictive type, the extent of the decrease in the serum phosphorus level and in the serum potassium level was 1.2 ± 1.7 mg/dl and 0.6 ± 0.5 mmol/l, respectively. In this group again, a moderate positive correlation was found between the two electrolytes (r = 0.33, p = 0.03). The extent of the decrease in the serum phosphorus level and in the serum potassium level for the binge–purge type was 1.0 ± 1.1 mg/dl and 0.1 ± 0.2 mmol/l, respectively. The correlation between the two did not reach statistical significance (p = 0.84).

## Discussion

Our present study showed that binge–purge behavior, a lower body mass index, and hypoalbuminemia are associated with a lower serum potassium level at admission and a lower nadir potassium level during refeeding in patients with anorexia nervosa. Although some of these three indicators did not reach statistical significance in the sub-analyses, this might have resulted from low statistical power, and thus these variables might reach statistical significance in a large-scale study. These findings suggest that these factors are useful for predicting hypokalemia and when to consider potassium supplementation for patients with anorexia nervosa. To our knowledge, except for binge–purge behavior, this is the first report on predictors for hypokalemia that are associated with anorexia nervosa during the refeeding period.

Our study found that malnutrition and morbidity, which are represented by a lower body mass index and hypoalbuminemia [23], respectively, significantly contributed to hypokalemia at admission in patients with anorexia nervosa. This was also the case with hypokalemia during refeeding in our study, which is quite similar to the fact that malnutrition and morbidity causes refeeding hypophosphatemia in patients with anorexia [24, 26, 30, 31]. In fact, there was a positive correlation between the extent of the decrease in serum potassium and phosphorus levels in our study, suggesting that reintroduction of nutrients led to the intracellular movement of serum potassium and phosphorus, the hallmark of refeeding syndrome. The fact that there was no correlation between hypophosphatemia and hypokalemia during refeeding for the binge–purge type is considered to be attributed to more supplemental potassium administration to these patients than that to patients with restrictive type. Still, even among individuals in the binge–purge group, a lower serum potassium level at admission was predicted by a lower body mass index, suggesting that not only binge–purge behavior but malnutrition lead to hypokalemia even in this cohort.

The fact that a higher caloric intake during the refeeding period was associated with a higher nadir potassium level during this same time period might indicate that patients were able to take in potassium to a certain extent via their meals or liquid formulas when the composition of those nutrients included potassium. This is consistent with the findings on caloric intake for refeeding hypophosphatemia, in which a higher caloric intake was not correlated with the development of refeeding hypophosphatemia or with a decrease in the serum phosphorus level [26, 31, 35–38]. Of note, lower calorie intake after admission was associated with a lower nadir potassium level in patients with restrictive type, who had a significantly low body mass index at an average of 12.3 kg/m^2^. These results suggest that a higher-calorie diet containing potassium might alleviate the decrease of serum potassium levels in patients with extremely severe malnutrition.

Although hypomagnesemia is known to cause refractory hypokalemia [16, 17], our study found the negative correlation of albumin-corrected magnesium level and serum potassium level. This might be partly explained by prehospital magnesium oxide administration, which elevated albumin-corrected magnesium levels and was used mainly by patients with the binge–purge type, who had a lower serum potassium levels, although the exact number of patients who had actually took magnesium oxide might have been higher because magnesium oxide can be obtained without a prescription. In fact, hypermagnesemia (3.2 ± 1.1 mg/dl) was found in patients who had been prescribed with magnesium oxide before being hospitalized. If a patient secretly overused both magnesium oxide and a diuretic at the same time, these drugs should have caused the patient to have both hypermagnesemia and hypokalemia. Considering these unique situations in patients with anorexia nervosa, in addition to binge–purge behavior, a lower body mass index, and hypoalbuminemia, a structured interview with regards to laxative/diuretic abuse is needed to precisely investigate serum potassium and magnesium levels.

## Limitations

Our study has several limitations that should be considered. First, the amount of oral potassium intake was not investigated because we often allowed patients to bring their favorite food from home to increase their appetite, and the amount of potassium in such food was not able to be precisely calculated. Second, absence of a standardized protocol for potassium administration might represent a limitation in study design and interpretation of the data analyses. However, the actual amount of potassium supplementation was controlled for our multivariable regression analyses. Third, not all factors that might affect serum potassium levels were taken into account, such as metabolic acidosis due to peripheral circulatory insufficiency associated with extreme malnutrition. Fourth, although we tried to investigate serum potassium levels frequently in each patient, ideally these levels should be examined every day during the refeeding period to precisely determine the nadir serum potassium level. Fifth, although treatment-related indicators were measured during the first week, period of the first 2 weeks was applied for the lowest potassium levels. Indeed, nadir hypokalemia was observed during the second week in 11 admissions (16.6%). This is a difficult issue to address because if 1 week was applied for nadir hypokalemia, the actual level of nadir hypokalemia was unable to be measured. It is also difficult to apply 2 week period for treatment-related indicators because patients had already been discharged from hospital within 2 weeks after admission in 8 admissions (12.1%). Sixth, this study was performed over a period of 15 years. Some treatment strategies might have changed, especially adapting of a higher-calorie diet during the refeeding period [35–39]. However, calorie intake was controlled in our multivariable regression analyses. Seventh, the generalizability of our results is limited because our study population was derived from a single hospital. Finally, the study population was not large, especially with respect to the sub-analyses based on the binge–purge or restrictive type. These issues should be addressed in future studies.

## Conclusions

Despite the aforementioned limitations, our study demonstrates that a lower body mass index, hypoalbuminemia, and binge–purge behavior might predict a lower serum potassium level among patients with anorexia nervosa. These factors may be useful indicators for controlling serum potassium levels for inpatients with anorexia nervosa.

## Data Availability

The datasets generated and/or analyzed during the current study are available from the corresponding author (MF) upon request.

## References

[1] Hofer M, Pozzi A, Joray M, Ott R, Hähni F, Leuenberger M, von Känel R, Stanga Z (2014). Safe refeeding management of anorexia nervosa inpatients: an evidence-based protocol. Nutrition.

[2] Mehler PS, Blalock DV, Walden K, Kaur S, McBride J, Walsh WJ (2018). Medical findings in 1,026 consecutive adult inpatient-residential eating disordered patients. Int J Eating Disorders.

[3] Guinhut M, Melchior JC, Godart N, Hanachi M (2020). Extremely severe anorexia nervosa: Hospital course of 354 adult patients in a clinical nutrition-eating disorders-unit. Clin Nutr.

[4] Bonne OB, Bloch M, Berry EM (1993). Adaptation to severe chronic hypokalemia in anorexia nervosa: a plea for conservative management. Int J Eat Disord.

[5] Krantz MJ, Blalock DV, Tanganyika K, Farasat M, McBride J, Mehler PS (2020). Is QTc-interval prolongation an inherent feature of eating disorders? A cohort study. Am J Med.

[6] Stokke A, Julsrud J, Fosse A, Nielsen EW (2011). A young woman with anorexia, hypokalemia and convulsion. Tidsskr Nor Laegeforen.

[7] Finsterer J, Stöllberger C (2014). Recurrent aborted sudden cardiac death with seizures and rhabdomyolysis due to bulimia-induced hypokalemia: report of one case. Rev Med Chil.

[8] Krahn LE, Lee J, Richardson JW, Martin MJ, O'Connor MK (1997). Hypokalemia leading to torsades de pointes. Munchausen's disorder or bulimia nervosa?. Gen Hosp Psychiatry.

[9] Lee EY, Yoon H, Yi JH, Jung WY, Han SW, Kim HJ (2015). Does hypokalemia contribute to acute kidney injury in chronic laxative abuse?. Kidney Res Clin Pract.

[10] Yasuhara D, Naruo T, Taguchi S, Umekita Y, Yoshida H, Nozoe S (2005). “End-stage kidney” in longstanding bulimia nervosa. Int J Eat Disord.

[11] Choi JW, Kwon SK, Kim SM, Cho H, Lee HC, Kim HY (2018). Interstitial nephritis caused by anorexia nervosa in young male; a case report and literature review. Electrolyte Blood Press.

[12] Greenfeld D, Mickley D, Quinlan DM, Roloff P (1995). Hypokalemia in outpatients with eating disorders. Am J Psychiatry.

[13] Cheungpasitporn W, Suksaranjit P, Chanprasert S (2012). Pathophysiology of vomiting-induced hypokalemia and diagnostic approach. Am J Emerg Med.

[14] Vavruk AM, Martins C, Nascimento MM, Hayashi SY, Riella MC (2012). Association between hypokalemia, malnutrition and mortality in peritoneal dialysis patients. J Bras Nefrol.

[15] Raza M, Kumar S, Ejaz M, Azim D, Azizullah S, Hussain A (2020). Electrolyte imbalance in children with severe acute malnutrition at a tertiary care hospital in Pakistan: a cross-sectional study. Cureus.

[16] Hall RC, Hoffman RS, Beresford TP, Wooley B, Tice L, Hall AK (1988). Refractory hypokalemia secondary to hypomagnesemia in eating disorders patients. Case Reports Psychosomatics.

[17] Huang CL, Kuo E (2007). Mechanism of hypokalemia in magnesium deficiency. J Am Soc Nephrol.

[18] Grasso S, Ferro Y, Migliaccio V, Mazza E, Rotundo S, Pujia A, Montalcini T (2013). Hypokalemia during the early phase of refeeding in patients with cancer. Clinics (Sao Paulo).

[19] Garber AK, Sawyer SM, Golden NH, Guarda AS, Katzman DK, Kohn MR, Le Grange D, Madden S, Whitelaw M, Redgrave GW (2016). A systematic review of approaches to refeeding hospitalized patients with anorexia nervosa. Int J Eat Disord.

[20] Mehanna HM, Moledina J, Travis J (2008). Refeeding syndrome: what it is, and how to prevent and treat it. BMJ.

[21] Crook MA, Hally V, Panteli JV (2001). The importance of the refeeding syndrome. Nutrition.

[22] Miller SJ (2008). Death resulting from overzealous total parenteral nutrition: the refeeding syndrome revisited. Nutr Clin Pract.

[23] Marcason W (2017). Should albumin and prealbumin be used as indicators for malnutrition?. J Acad Nutr Diet.

[24] Redgrave GW, Coughlin JW, Schreyer CC, Martin LM, Leonpacher AK, Seide M, Verdi AM, Pletch A, Guarda AS (2015). Refeeding and weight restoration outcomes in anorexia nervosa: challenging current guidelines. Int J Eat Disord.

[25] Kameoka N, Iga J, Tamura M, Tominaga T, Kubo H, Watanabe Y, Sumitani S, Tomotake M, Ohmori T (2016). Risk factors for refeeding hypophosphatemia in Japanese inpatients with anorexia nervosa. Int J Eat Disord.

[26] Funayama M, Mimura Y, Takata T, Koreki A, Ogino S, Kurose S (2021). Body mass index and blood urea nitrogen to creatinine ratio predicts refeeding hypophosphatemia of anorexia nervosa patients with severe malnutrition. J Eat Disord.

[27] Ornstein RM, Golden NH, Jacobson MS, Shenker IR (2003). Hypophosphatemia during nutritional rehabilitation in anorexia nervosa: implications for refeeding and monitoring. J Adolesc Health.

[28] Kroll MH, Elin RJ (1985). Relationships between magnesium and protein concentrations in serum. Clin Chem.

[29] Fein P, Weiss S, Ramos F, Singh P. Serum magnesium concentration is a significant predictor of mortality in peritoneal dialysis patients. In: Advances in peritoneal dialysis. Conference on peritoneal dialysis 2014; 30: 90–3. https://www.advancesinpd.com/adv14/90-93_Fein.pdf 25338428

[30] Golden NH, Keane-Miller C, Sainani K, Kapphahn CJ (2013). Higher caloric intake in hospitalized adolescents with anorexia nervosa is associated with reduced length of stay and no increased rate of refeeding syndrome. J Adolesc Health.

[31] Brown CA, Sabel AL, Gaudiani JL, Mehler PS (2015). Predictors of hypophosphatemia during refeeding of patients with severe anorexia nervosa. Int J Eat Disord.

[32] Macedo E, Mehta R. Clinical Approach to the Diagnosis of Acute Kidney Injury. National Kidney Foundation Primer on Kidney Diseases (Sixth Edition) (Gilbert S, Weiner D, Eds.) 2014, 294–303. https://www.elsevier.com/books/national-kidney-foundation-primer-on-kidney-diseases/9781455746170

[33] Monteleone AM, Monteleone P, Serino I, Amodio R, Monaco F, Maj M (2016). Underweight subjects with anorexia nervosa have an enhanced salivary cortisol response not seen in weight restored subjects with anorexia nervosa. Psychoneuroendocrinology.

[34] Morino K, Kondo K, Tanaka S, Nishida Y, Nakae S, Yamada Y, Ugi S, Fuse K, Miyazawa I, Ohi A, Nishida K, Kurihara M, Sasaki M, Ebine N, Sasaki S, Katsukawa F, Hiroshi M. Total energy expenditure is comparable between patients with and without diabetes mellitus: Clinical Evaluation of Energy Requirements in Patients with Diabetes Mellitus (CLEVER-DM) Study. BMJ Open Diabetes Res Care 2019; 7, e000648. 10.1136/bmjdrc-2019-000648 eCollection 2019. 10.1136/bmjdrc-2019-000648 PMC6501857 31114702

[35] Whitelaw M, Gilbertson H, Lam PY, Sawyer SM (2010). Does aggressive refeeding in hospitalized adolescents with anorexia nervosa result in increased hypophosphatemia?. J Adolesc Health.

[36] Garber AK, Michihata N, Hetnal K, Shafer MA, Moscicke AB (2012). A prospective examination of weight gain in hospitalized adolescents with anorexia nervosa on a recommended refeeding protocol. J Adolesc Health.

[37] O’Connor G, Nicholls D (2013). Refeeding hypophosphatemia in adolescents with anorexia nervosa: a systematic review. Nutr Clin Pract.

[38] Garber AK, Mauldin K, Michihata N, Buckelew SM, Shafer MA, Moscicki AB (2013). Higher calorie diets increase rate of weight gain and shorten hospital stay in hospitalized adolescents with anorexia nervosa. J Adolesc Health.

[39] Katzman DK, Garber AK, Kohn M, Golden NH (2014). Refeeding hypophosphatemia in hospitalized adolescents with anorexia nervosa. J Adolesc Health.

